# Density-dependent signaling: An alternative hypothesis on the function of chemical signaling in a non-territorial solitary carnivore

**DOI:** 10.1371/journal.pone.0184176

**Published:** 2017-10-05

**Authors:** Clayton T. Lamb, Garth Mowat, Sophie L. Gilbert, Bruce N. McLellan, Scott E. Nielsen, Stan Boutin

**Affiliations:** 1 Department of Biological Sciences, University of Alberta, Edmonton, Alberta, Canada; 2 Ministry of Forests Lands and Natural Resource Operations, Nelson, British Columbia, Canada; 3 Department of Earth, Environmental and Geographic Sciences, The University of British Columbia Okanagan Campus, Kelowna, British Columbia, Canada; 4 Department of Renewable Resources, University of Alberta, Edmonton, Alberta, Canada; Université de Sherbrooke, CANADA

## Abstract

Brown bears are known to use rubbing behavior as a means of chemical communication, but the function of this signaling is unclear. One hypothesis that has gained support is that male bears rub to communicate dominance to other males. We tested the communication of dominance hypothesis in a low-density brown bear population in southeast British Columbia. We contrasted rubbing rates for male and female bears during and after the breeding season using ten years of DNA-mark-recapture data for 643 individuals. Here we demonstrate that male brown bears rub 60% more during the breeding than the non-breeding season, while female rubbing had no seasonal trends. Per capita rub rates by males were, on average, 2.7 times higher than females. Our results suggest that the function of rubbing in the Rocky Mountains may not only be to communicate dominance, but also to self-advertise for mate attraction. We propose that the role of chemical communication in this species may be density-dependent, where the need to self-advertise for mating is inversely related to population density and communicating for dominance increases with population density. We suggest that future endeavors to elucidate the function of rubbing should sample the behavior across a range of population densities using camera trap and genotypic data.

## Introduction

Chemical communication within and amongst species can provide direct fitness benefits by reducing predation risk [1,2], competition [3], physical conflict [4], and facilitating mate attraction [5]. Brown bears (*Ursus arctos*) are known to chemically communicate by rubbing on trees, which can be sampled using non-invasive methods such as camera traps or genotyping of hairs left on the tree during rubbing [6–8]. Clapham et al. (2012) [6] proposed and tested competing hypotheses on the function of chemical signaling among brown bears. These hypotheses included: 1) self-advertisement for mate attraction, 2) communication of dominance, 3) competitor assessment, and, 4) infanticide avoidance. These researchers found evidence for the communication of dominance hypothesis, especially between males, and proposed this as the primary function of chemical signaling in the species.

**Fig 1:**
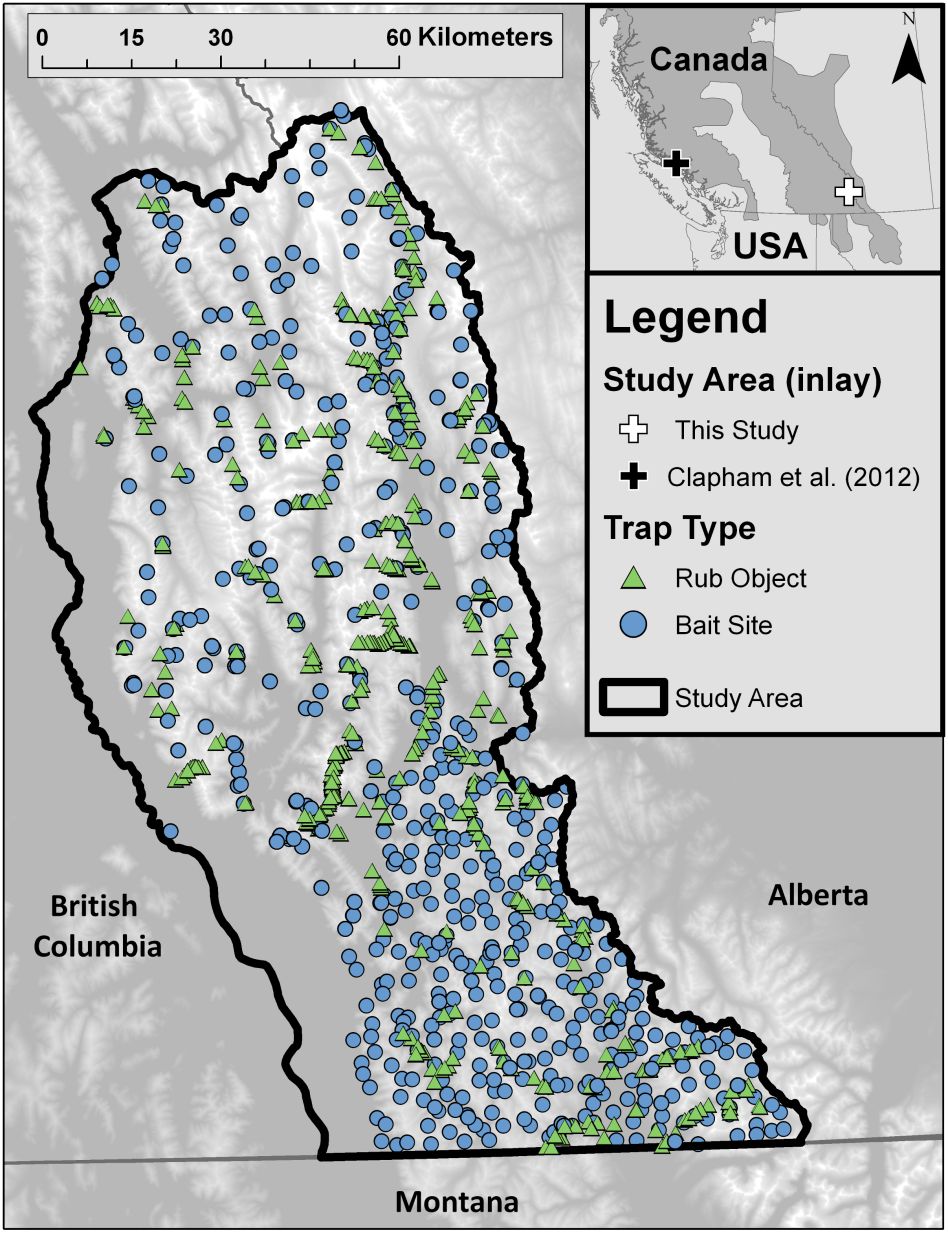
Study area and site locations for ten years of DNA mark-recapture sampling (2006–2015) conducted for grizzly bear population monitoring in southeast British Columbia, Canada. Elevation shown as basemap, where low elevation (min = 442 m) is shown in dark grey, and high elevation (max = 3930 m) in white. Location of this and Clapham et al. (2012) study as well as southern grizzly distribution in North America (dark grey) shown in inset map.

The generality of the communication of dominance hypothesis has yet to be investigated across different ecosystems or bear population densities. Clapham et al.’s 2012 study took place in a small study area (~15 km^2^ [Clapham pers. comm]) in Glendale Cove, British Columbia (BC) where concentrated resources (salmon in the Glendal River and abundant vegetation in estuaries) produce high local densities of brown bears ([9]; Fig 1). Visual observations of bears by Clapham et al. (2012) suggested that a maximum of 21–52 individual bears (>1000 bears / 1000 km^2^) were detected in this region seasonally, a local density 20-40x higher than reported for brown bear populations in the interior mountainous regions of North America where food resources are much more dispersed [10]. We were unable to find a population density estimate for the larger population around Glendale Cove, but brown bear populations with access to concentrated food resources such as salmon often occur at much higher population densities than those without [11]. It is not known if the findings of Clapham et al. (2012) are generalizable to local densities more typical for this species (10–200 bears / 1000 km^2^).

Clapham et al. concluded that the chemical signaling in the Glendale Cove was primarily for mate attraction, because males continued to mark (rub, bite, claw or urinate on) trees at a high level post-breeding. This behavior, which they concluded was aimed at communicating dominance, may have been motivated by the high density of bears, and also by an influx of new bears attracted to spawning salmon after the breeding season; creating a very high local density of bears not typically observed across much of the species range. Several other studies on brown bears living at lower densities, and without seasonal immigration, have reported that the highest rate of rubbing on trees and other objects is by males during the breeding season [12–17]; suggesting the temporal pattern of rubbing, and thus function, may differ with population density. Using a 10-year dataset, we had the opportunity to test whether there was more support for the mate attraction hypothesis in a population of brown bears in the Rocky Mountains of British Columbia existing at a density (20–80 bears/1000 km2) [10,18], at least an order of magnitude lower than in Clapham et al.’s (2012) study.

## Methods

### Ethical statement

This project was approved and sponsored by the Province of British Columbia, Ministry of Forests, Lands and Natural Resource Operations, prior to the first year of sampling (2006). DNA-based mark–recapture methods used in this study for grizzly bears are exempt from capture permits in British Columbia. Ethical approval for the analyses of these data was provided by the University of Alberta Research Ethics Office, December 2014. The authors have no conflicts of interest to disclose.

### Study area

Our region of investigation in southeast British Columbia covered a large geographic extent (12,000 km^2^, Fig 1) in the Rocky Mountains where food resources are more spatially dispersed than in Glendale Cove [19]. Approximately 350 bears occupy the study area [19] and feed primarily on forbs, roots, grasses, fruits, and ungulates when available [20]. Brown bears in the Rocky Mountains consume the most herbivorous diet of the North America population [21] and bears in the Rocky Mountains are typically smaller and fatter than bears that consume more meat-based protein [22]. Most valleys in our study area have at least one road or linear feature in the valley bottom for industrial activities such as logging, coal mining, or mineral and gas exploration.

### Study design

We sought to compare the frequency of rubbing behaviour at rub objects (mostly trees, but some power poles or fence posts where bears naturally rub), and thus investment in chemical signalling, by sex (male and female) and season (breeding [June 1-July 31] and non-breeding [Aug 1- Oct 5]). Although the bulk of breeding activity appears to be earlier in July [23], we maintained the same dates as Clapham et al. (2012) for comparative purposes and because post-copulatory behavior may last until the end of July [24]. We wrapped rub objects with barbed wire to increase the amount of hair collected and to reduce samples with mixed DNA; as is standard practice [7]. Increased rubbing by either sex during the breeding season (compared to the non-breeding season) would support the ‘mate attraction’ hypothesis, while more sustained investment in rubbing through both the breeding and non-breeding seasons would support the ‘communicating dominance’ hypothesis (Table 1). We contrasted detection rates between rub objects and baited hair snag sites (bait sites [25,26]). Bait sites rely on a scented lure (rotten cow blood and fish oil) to attract bears to a hair collection device in hopes of finding a food reward, whereas rub objects rely on the innate behavioral motivation of rubbing and communication. Comparing detections at rub objects and bait sites between seasons allowed us to isolate the behavioral response of bears at rub objects. For example, if bait site detection is static between seasons while rub object detection varies, we can attribute this variation to the behavioral response at rub objects and not a change in detection rates due movement rates [27] given that bait sites and rub objects are both sampled across the entire landscape (Fig 1).

**Table 1:** Hypotheses and predictions, adapted from Clapham *et al*. (2012) and adapted to the data from this study. We were unable to test two of the hypotheses proposed by Clapham *et al*. (2012) (Competitor Assessment and Infanticide Avoidance), as well as some of the predictions that involved information on investigatory or age-class information. M = Male, F = Female. BS = Breeding Season, NON-BS = Non-Breeding Season, Y = Yes, N = No, P = Partially. We were unable to determine adult from non-adult in our work, thus where Clapham *et al*. (2012) distinguish adult and subaduls, we simply pool these groups into their respective sexes but not age classes.

Hypothesis	Prediction	Prediction supported?	Hypothesis supported?
1. Self-advertisement for mate attraction			
1.1 AM self-advertise	1.1.1 M will scent mark at a higher frequency during the BS than NON-BS	Y	Y
1.2 AF self-advertise	1.2.1 F will scent mark at a higher frequency during the BS than NON-BS	N	N
2. Communicating dominance			
2.1 AM communicate dominance	2.1.1 M will scent mark at similar frequencies during BS and NON-BS	N	P
	2.1.2 M will scent mark at a frequency higher than F in both the BS and NON-BS	Y	
2.2 AF do not communicate dominance	2.2.1 F will scent mark at similar frequencies during BS and NON-BS	Y	P
	2.2.2 F will scent mark at a frequency higher than M in both the BS and NON-BS	N	

We tested two of the four hypotheses set out by Clapham *et al*. (2012) (Table 1), but lacked camera trap data which generate age-class and investigatory behaviour, precluding our ability to test the remaining two hypotheses (competitor assessment and infanticide avoidance). We used the predictions set out by Clapham *et al*. (2012), for which our data was appropriate for, to test the communication of dominance and self-advertisement for mates hypotheses. Clapham *et al*. (2012) compared rubbing rates of bears to their sex age-class prevalence in the local population for each season (breeding and non-breeding) due to seasonal influx of bears into the study area. Unlike Clapham *et al*. (2012), our study is not as sensitive to seasonal immigration because the study area is large enough to encompass seasonal movements of bears between food sources. Thus in Table 1 where Clapham *et al*. (2012) compared the frequency of breeding and non-breeding to an expected distribution, we compare breeding and non-breeding to each other. We infer that bears are rubbing (marking) at a frequency different than expected if rubbing rates in the breeding and non-breeding seasons are unlikely to be the same (using p values).

### Data collection

We determined detection rates of brown bears by sex, season (breeding and non-breeding) and detector (rub object or bait site) using DNA mark-recapture data across a 12,000 km^2^ area in southeast British Columbia (Fig 1; Lamb et al. 2016a). During 2006–2015 we collected 11,430 hair samples from 1,897 sites (counted as site-year combinations) that were visited between June-Oct for a cumulative 145,284 trap nights during our ten years of study (Table 2). Bait sites were generally deployed in the breeding season and usually checked every two weeks. We sampled into the non-breeding season in 2012, allowing us to investigate detection success in both breeding and non-breeding seasons. In most years rub objects were sampled in both the breeding and non-breeding seasons (Table 2) and were visited at least once per month. As discussed in more detail below, we included a random effect of year in our models to account for annual changes in population size, portion of study area sampled, and duration of sampling.

**Table 2:** Annual detection success for grizzly bears in southeast British Columbia, Canada between 2006–2015. Detections represent the number of hair samples genotyped to an individual, and individuals detected is the number of unique individuals detected in each trap-type each year.

Year	Trap	Sites	Trap Nights	Start	End	Hair Samples	Detections	Individuals Detected	Males	Females
**2006**	Bait Site	68	1916	Jun-11	Jul-28	1000	106	88	40	48
	Rub Object	5	128	Jun-11	Oct-05	15	3	3	3	0
**2007**	Bait Site	71	1709	Jun-13	Jul-20	1075	105	97	40	57
	Rub Object	16	596	Jun-01	Oct-18	54	8	6	4	2
**2008**	Bait Site	83	1938	Jun-25	Jul-31	713	87	81	34	47
	Rub Object	44	1439	May-14	Oct-02	224	25	19	14	5
**2009**	Bait Site	57	1531	Jun-24	Jul-24	757	96	80	39	41
	Rub Object	124	5072	Jun-10	Sep-25	568	64	48	38	10
**2010**	Bait Site	27	762	Jun-22	Jul-25	297	47	40	20	20
	Rub Object	169	7465	May-18	Oct-26	417	62	52	34	18
**2011**	Bait Site	22	579	Jun-29	Jul-29	133	28	27	14	13
	Rub Object	177	12481	Jun-16	Oct-19	545	93	66	45	21
**2012**	Bait Site	16	1985	Jun-04	Oct-07	660	87	35	19	16
	Rub Object	190	21197	Jun-05	Oct-15	840	169	109	66	43
**2013**	Bait Site	52	1480	Jun-27	Jul-29	366	53	49	22	27
	Rub Object	238	20487	Jun-13	Oct-11	854	135	110	59	51
**2014**	Bait Site	42	1151	Jun-25	Jul-29	502	60	52	20	32
	Rub Object	242	25314	May-22	Oct-17	1079	167	101	62	39
**2015**	Bait Site	-	-	-	-	-	-	-	-	-
	Rub Object	254	38054	May-21	Oct-27	1331	224	127	72	55
**2006–2015**	**Bait Site**	**438**	**13051**			**5503**	**832**	**474**	**214**	**260**
**2006–2015**	**Rub Object**	**1459**	**132233**			**5927**	**950**	**343**	**187**	**156**

Hair samples were genotyped by Wildlife Genetics International in Nelson, British Columbia. We sub-sampled hair samples based on our previous work [28] suggesting that we could reduce genotyping costs while still detecting most individuals. Sub-sampling procedures included removing samples that were unlikely to produce a genotype (too few hairs, mixed individuals, low DNA quality), were unlikely to be a grizzly bear and finally were simply removed as per our sub-sampling procedures to reduce the number of duplicate identifications of an individual at a site [28,29]. Using multilocus (9 loci, [30]) genotyping of hair samples, we detected 643 (336 F, 307 M) individual bears a total of 1782 times [31]. Unlike previous investigations into the function of rubbing [6,16], we lacked the age-class of bears rubbing and investigatory behavior and instead use population-level inferences to provide evidence for the function of scent marking by individuals of known identity and sex.

For each year, we calculated a sex-specific measure of relative detection success (RDS, [29]) using the average daily detection rate across all traps (average # of bears per trap), which we then scaled by a sex-specific density (calculated using spatial capture-recapture methods [32]). Finally, we calculated RDS per 1000 trap nights (RDS1000) for easier comparison between values with whole numbers.

To investigate differences in relative detection success between bait sites and rub sites, we used a linear mixed-effects model with a random effect for year (2006–2015) using the ‘lme4’ package in program R. The response variable was relative detection success (# of bears detected / 1000 trap nights, RDS1000), while predictive variables included sex, detector type (bait site vs. rub object) and season: breeding and non-breeding. We built a fully parametrized model (RDS1000~ Season + Trap Type + Sex + Season*Trap Type*Sex + Random effect of year) and used the ‘multcomp’ package in R to determine the p-values using Tukey’s all-pair comparisons. All assumptions for statistical tests were met.

## Results

**Fig 2:**
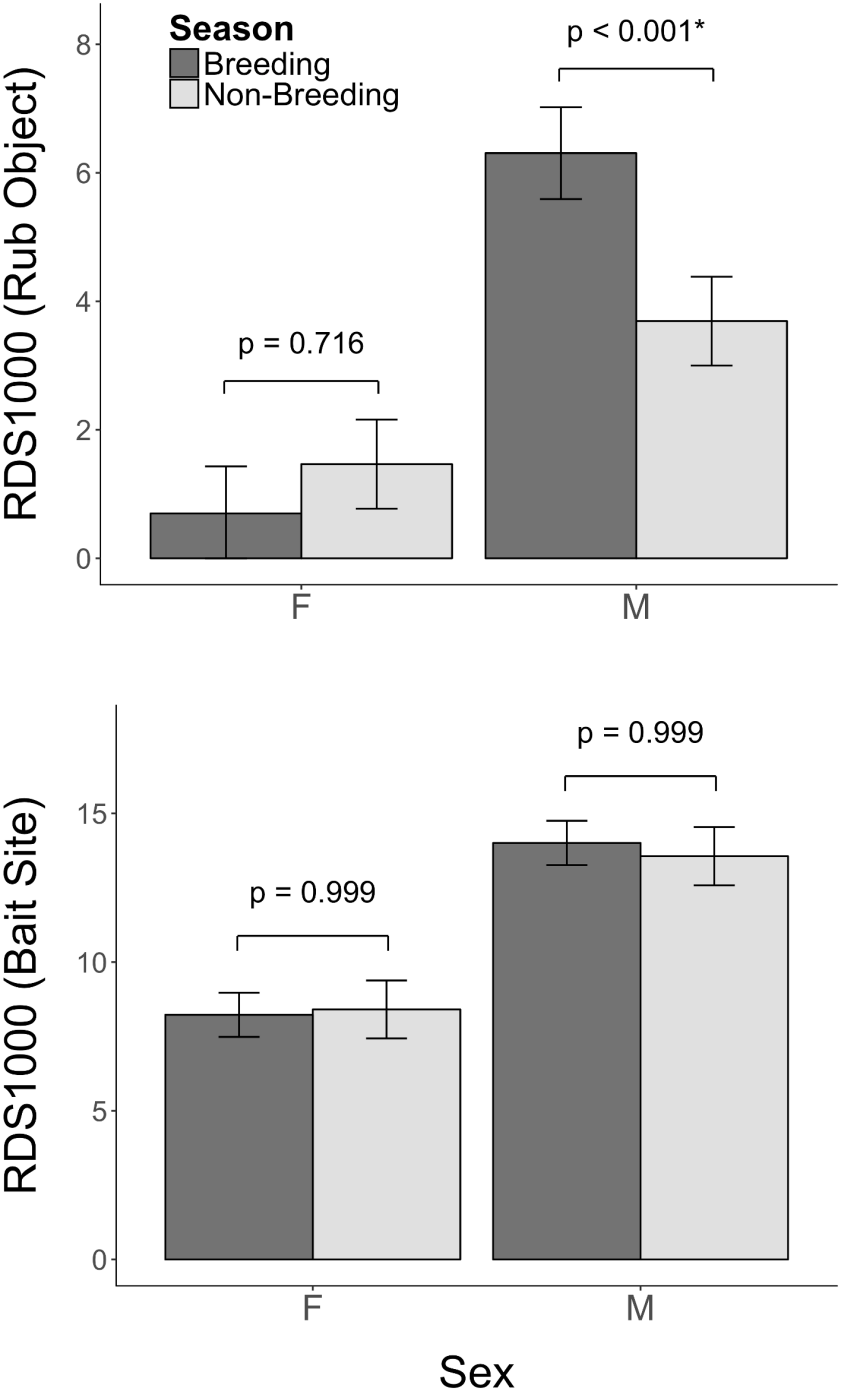
Relative detection success (RDS1000, average daily detection rate per 1000 trap nights for each sex (F = female, M = male) by trap-type and scaled by sex-specific density) for DNA mark-recapture data for brown bears from southeast British Columbia, Canada. Seasons follow Clapham et al. (2012) where breeding is June 1-July 31, and non-breeding is Aug 1- Oct 5. Rub object [top] and bait site comparison [bottom]. Error bars are standard errors.

Males rub marked on rub objects significantly more (*p* < 0.001, Fig 2) than female bears in both the breeding (RDS1000: Male = 5.5 ± 7.6 (SE); Female = 1.0 ± 7.7) and non-breeding seasons (RDS1000: Male = 3.3 ± 7.4; Female = 1.4 ± 7.4). Male bears rub marked on rub objects significantly less (41% less) during the non-breeding versus the breeding season (*p* < 0.001, Fig 2), whereas females showed no seasonal differences (*p* = 0.716, Fig 2). We did not detect a similar decline in detection of males or females at bait sites after the breeding season (*p* = 0.999, Fig 2) suggesting that the decline in male rubbing after the breeding season reflected a behavioral difference rather than a change in detection due to changes in seasonal movement rates. Generally, relative detection rates were higher for males and bait sites (Table 3 & Fig 2).

**Table 3:** Coefficients from the full model for relative detection success (RDS1000). Categorical variables are relative to their categorical base values which were were set as follows: Season = Non-Breeding, Trap Type-Rub Object, and Sex = Male.

Variable	Estimate	Std. Error	Lower 95% CI	Upper 95% CI
Intercept	0.00823	0.00074	0.00677	0.00968
Season	0.00018	0.00089	-0.00157	0.00193
Trap Type	-0.00753	0.00068	-0.00886	-0.00619
Sex	0.00578	0.00062	0.00457	0.00699
Season * Trap Type	0.00059	0.00103	-0.00143	0.00261
Season * Sex	-0.00063	0.00125	-0.00307	0.00182
Trap Type * Sex	-0.00017	0.00083	-0.00180	0.00146
Season * Trap Type * Sex	-0.00275	0.00144	-0.00557	0.00006

## Discussion

Our results suggest a modified interpretation of the communication of dominance hypothesis posited by Clapham et al. (2012) for chemical signaling of brown bears, a non-territorial solitary carnivore. Consistent with the communication of dominance hypothesis, male bears in the southern Rockies rub mark more than females in both the breeding and non-breeding seasons. However, unlike Clapham et al. (2012), we detected a large decline in male rub marking after the cessation of the breeding season, providing support for one of Clapham et al’s alternative hypotheses: that male brown bears chemically signal to self-advertise for mate attraction.

Female brown bears may seek out males during the breeding season as evidenced by spatial associations of collared individuals (Stenhouse, unpublished data). We demonstrate that male brown bears in our study system, living at much lower local densities than those studied by Clapham et al. (2012), likely engage in rub mark behavior to communicate dominance and self-advertise for mates, suggesting that these hypotheses are not mutually exclusive [16]. Marking by males during and after the breeding season (although at a reduced rate) may also suggest that self-advertising would enhance female choice during the current and following breeding seasons, particularly in low-density populations.

Male bears living in low-density populations should receive fitness benefits by increasing their advertisements during the breeding season; given males have the ability to breed with multiple females per year. But, because bears are not territorial, and males may encounter one another in their home range [24], male bears must maintain a level of rubbing throughout the entire active season to establish and maintain dominance hierarchies and thus reduce costly physical encounters, or to maintain social status for the following breeding season [6]. We note that it is difficult to tease apart the degree of breeding season rubbing attributable to self-advertisement versus elevated dominance communication with our data. Given that both intra- and inter-sexual interactions should be less frequent at lower population density (e.g. in our population), the limiting factor to an individual’s fitness in this case appears to be the number of mates one can find, not avoiding costly intrasexual encounters. Consequently, we suggest that the increase in rubbing behavior during the breeding season in low-density populations is primarily due to males advertising for females, and to a lesser degree to communicate dominance to other males.

Consistent with our results, numerous other studies on brown bear populations living at low population and local densities across the world have reported increased detection of male bears at rub objects during the breeding season, compared to the non-breeding season [12–17]. Recent work on pedal marking (scent marking with the interdigital glands of the foot as a bear approaches a rub tree) shows that this is almost exclusively a male behavior, which peaks during the mating season [33]. Furthermore, rubbing appears to be an innate mechanism since bears living at extremely low densities above tree line in the Arctic of Canada will rub on a post when presented even though they rarely have a natural rubbing object available [34].

We propose that the differences between our work and that of Clapham et al. (2012) is likely density-dependence in sociality, such that the fitness benefits gained by self-advertisement is inversely related to the availability of mates within an individual’s home range, whereas the benefits of communicating dominance increases with density and thus probability of encountering conspecifics. Coastal grizzly bears often live in much higher local densities over short periods of time than interior bears due to large aggregations of food resources, mainly salmon [11,35]. We suggest that the inferences from Clapham et al.’s 2012 study on the coast of British Columbia be limited to high-density populations until further investigation on the function of chemical signaling can be conducted across a range of population densities. High local densities of bears increases testosterone in males and the potential for conflict, and thus there is significant individual benefit to conflict reduction via communicating dominance in such areas [6,35]. Likewise, there should be little benefit in self-advertisement to attract mates in high-density areas where encounters between sexes are common. In contrast, brown bears existing at low densities may need to invest in mate attraction strategies through rubbing because individuals are more sparsely distributed across the landscape and encounters are correspondingly infrequent. Indeed, Stenhouse et al. (2005) demonstrated that intrasexual encounters are extremely rare (13% of documented bear-bear encounters were male-male) during the breeding season for a low-density brown bear population on the eastern slopes of the Rocky Mountains, while male-female associations were common (78% of documented encounters).

We believe our investigation into the function of chemical signaling in a low-density brown bear population provides a valuable addition to the limited study on this topic in wild populations. Nevertheless, some limitations of our study include the lack of investigatory behavior and information on age-class of bears, both of which limited our ability to test the full suite of hypotheses laid out by Clapham et al. (2012). We provide support for males self-advertising for mate attraction, but were unable to determine that females were in fact investigating these advertisements; although previous works have consistently shown female investigatory behavior [6, 26]. We had limited information (a single year, 2012) for brown bear detections at bait sites during the non-breeding season. We have no reason to believe that this lack of information affected our conclusions because inter-annual and between-sex marking rates were consistent during the breeding season. A strength in our work is that we could test hypotheses at a much larger scale than Clapham et al. (2012) and thus our results were not sensitive to the within home-range movements to seasonal foods, as was the case in Clapham et al. (2012). We also could identify individuals and their sex with near certainty for 643 individuals using genetic approaches, whereas Clapham et al. (2012) attempted to do this visually using photos from remote cameras.

We propose that chemical communication in brown bears is a function of local densities, with differential fitness benefits attributable to marking for mating or dominance depending on the local densities within an individual’s home range. Future work would benefit from combining the DNA-based approach used here, where individual identity and sex is known with certainty, with the camera-based methods of Clapham et al. (2012) in which age-class, reproductive status, and investigatory behavior can be observed. We encourage investigators to test this hypothesis across a broad range of population densities using information on rub marking and investigating from bears of known identity, sex and age to assess the generality of this hypothesis.
